# Late Post-Dissection Dynamic Intermittent Malperfusion of the Aortic Arch in Association with a Rare Heterogenous LOX Gene Variation

**DOI:** 10.3390/jcm13040952

**Published:** 2024-02-07

**Authors:** Barbara Leclercq, Julien Bertolino, Alexandre Rossillon, Vlad Gariboldi, Sarah El Harake, François Silhol, Michel Bartoli, Bernard Vaisse, Axel Bartoli, Gabrielle Sarlon-Bartoli

**Affiliations:** 1 Vascular Medicine and Arterial Hypertension Departement, La Timone Hospital, CHU Timone, 264, rue Saint-Pierre, 13385 Marseille cedex 5, France; bertolino.julien@ap-hm.fr (J.B.); gabrielle.sarlon@ap-hm.fr (G.S.-B.); 2 Vascular Surgery Department, La Timone Hospital, CHU Timone, 264, rue Saint-Pierre, 13385 Marseille cedex 5, France; alexandre.rossillon@ap-hm.fr (A.R.);; 3 Department of Cardiac Surgery, La Timone Hospital, CHU Timone, 264, rue Saint-Pierre, 13385 Marseille cedex 5, France; 4 Radiology Department, La Timone Hospital, CHU Timone, 264, rue Saint-Pierre, 13385 Marseille cedex 5, France; 5 Center for CardioVascular and Nutrition Research (C2VN), Aix Marseille University, 13005 Marseille, France

**Keywords:** intermittent occlusion, aortic dissection, late complication, LOX variation

## Abstract

Late ischaemic consequences of type A aortic dissection are rare. We present a 6-year late complication of type A aortic dissection treated by Bentall surgery in a 41-year-old patient. The patient presented with several episodes of lipothymia associated with hypertensive attacks with anisotension, cervicalgia, hemicranial headache, abdominal pain and lower limb slipping initially on exertion and later at rest. On dynamic examination, we diagnosed an intermittent dynamic occlusion of the aortic arch and rare LOX gene variation, which is considered to be associated with aneurysm or dissection of the ascending aorta in young patients. Surgical treatment by replacement of the ascending aorta and the aortic arch with reimplantation of the brachiocephalic trunk (BcTr) allowed the symptoms to resolve.

## 1. Introduction

Type A aortic dissection is a surgical emergency with a high mortality rate, especially when it occurs outside the hospital [1]. The latest American College of Cardiology (ACC) and American Heart Association (AHA) recommendations for the management of type A aortic dissection in patients with extensive aortic root destruction, root aneurysm, or known genetic aortic disease are to replace the aortic root with a mechanical or biological valve conduit [2]. The most common late complications of type A aortic dissection are downstream extension of the dissection and aneurysmal progression [3]. Ischaemic complications are observed in the acute phase of dissection with malperfusion syndromes in different arterial territories and by different mechanisms (dynamic or static obstruction) [4].

To the best of our knowledge, no case of coarctation due to a rupture of the residual intimal flap downstream of the brachiocephalic trunk has been described. Herein, we present a case of late dynamic intermittent obstruction of the aortic arch.

## 2. Case Report

We report the case of a 41-year-old man with a history of smoking. He was urgently referred to the cardiac surgery department for suspected recurrent aortic dissection.

His main medical history included a type A aortic dissection complicated by an unknown ascending aortic aneurysm measuring 70 mm. He had undergone urgent treatment with a Bentall surgery 6 years earlier. The dissection of the ascending aorta extended from the aortic root to the innominate artery and was further complicated by severe aortic regurgitation on the native tricuspid valve.

Recent clinical symptoms began ten days ago with the onset of hemicranial headache, intra-auricular pulsatile sensation, and right lateralised tinnitus. A few days later, the patient began to experience several episodes of lipothimia, initially during low-intensity exercise such as walking uphill. The term lipothymia refers to a feeling of sudden and transient discomfort that may be accompanied by sweating or visual or auditory signs, for example, but without loss of consciousness. These episodes recurred on several occasions and were associated with severe abdominal pain with faecal impaction and lower limb weakness. These episodes lasted a few minutes on average and were not associated with loss of consciousness.

On arrival at the emergency department, a systolic aortic murmur on auscultation, abolition of the left radial pulse on palpation, and anisotension (corresponding to a systolic blood pressure difference of 20 mmHg between the two upper limbs) were noted. There were no associated neurological symptoms.

**Figure 1:**
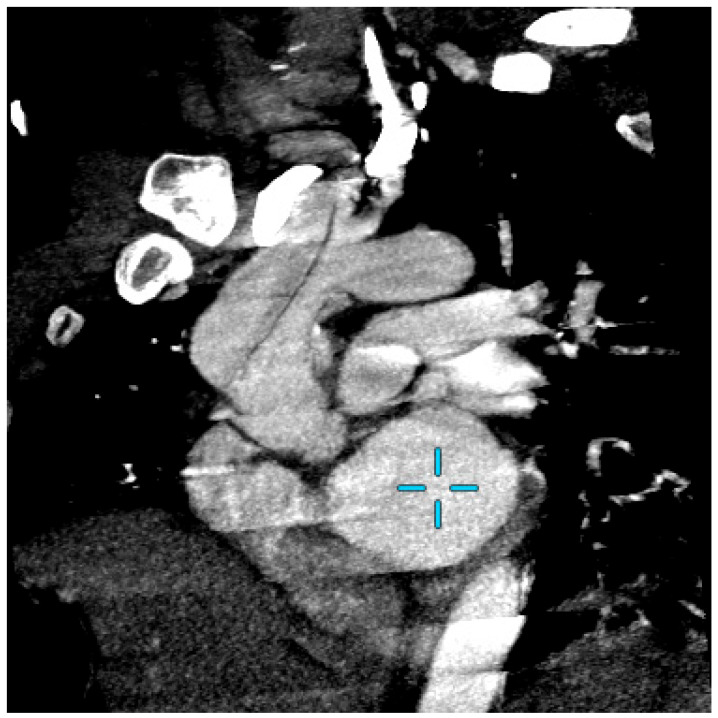
Computed tomography angiography (CTA) of aortic arch on emergency with “candy cane” aspect. The blue cross show the left ventricule.

Computed tomography angiography (CTA) excluded a recurrent aortic dissection and showed a residual valve opposite the brachiocephalic trunk without extension or residual aneurysm, which was unchanged from the results of a CTA scan carried out 5 years earlier (Figure 1).

The patient was admitted to cardiac surgery for further investigations. During hospitalisation, he underwent computed tomography angiography of the supra-aortic trunks, which showed no perfusion abnormalities.

Twenty-four-hour Holter blood pressure monitoring was performed and showed severe hypertensive peaks. It is important to note that the blood pressure cuff was placed on the right arm. In this context, antihypertensive treatment was initiated.

All of these examinations failed to reveal any evidence of a recurrence of aortic dissection and did not explain the symptoms described by the patient. Then, he was therefore transferred to the Vascular Medicine Department to have his arterial hypertension assessed, in particular to look for a cause of secondary hypertension such as pheochromocytoma. Medical therapy consisted of antihypertensive penta-therapy including a maximum-dose thiazide diuretic and curative anti-vitamin K anticoagulation.

Since the symptoms were paroxysmal, several examinations were carried out and repeated.

**Figure 2:**
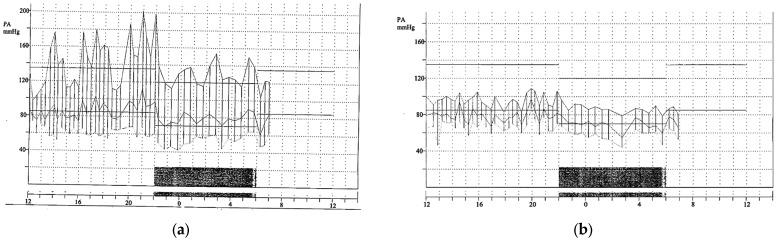
Twenty-four-hour Holter blood pressure monitoring on the right arm (**a**) and the left arm (**b**).

First, a new 24 h ambulatory blood pressure monitoring with simultaneous bilateral measurement of both upper limbs confirmed transient anisotension (Figure 2).

**Figure 3:**
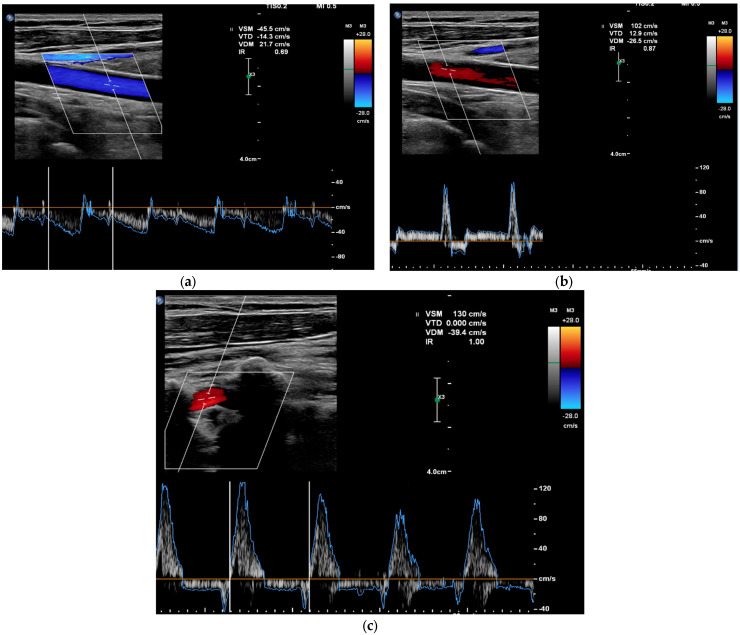
Colour and pulsed Doppler ultrasound on vascular Doppler ultrasound images. (**a**) Pre-critical left internal carotid artery with complete carotid flight. (**b**) Post-critical left internal carotid artery with intermittent carotid flight. (**c**) Post-critical left vertebral artery with intermittent carotid flight.

In addition, multiple vascular Doppler ultrasound (DUS) studies were performed. The first vascular Doppler study found no anatomical explanation for the anisotension. The second vascular Doppler ultrasound was performed during the critical phase of a lipothymic episode and showed a complete steal syndrome of the left carotid and vertebral arteries defined by a reversal of blood flow direction, with a contralateral hyperflow of the right carotid artery, which stopped after syncope (Figure 3). On colour and pulsed Doppler, there was a reversal in the direction of flow (antidromic) of the left internal carotid and vertebral arteries during the symptoms. At the end of the episode, the pulsed and colour Doppler analysis showed an intermittent flight of the left internal carotid artery and of the vertebral arteries defined by a reversal of the direction of blood flow in diastole.

**Figure 4:**
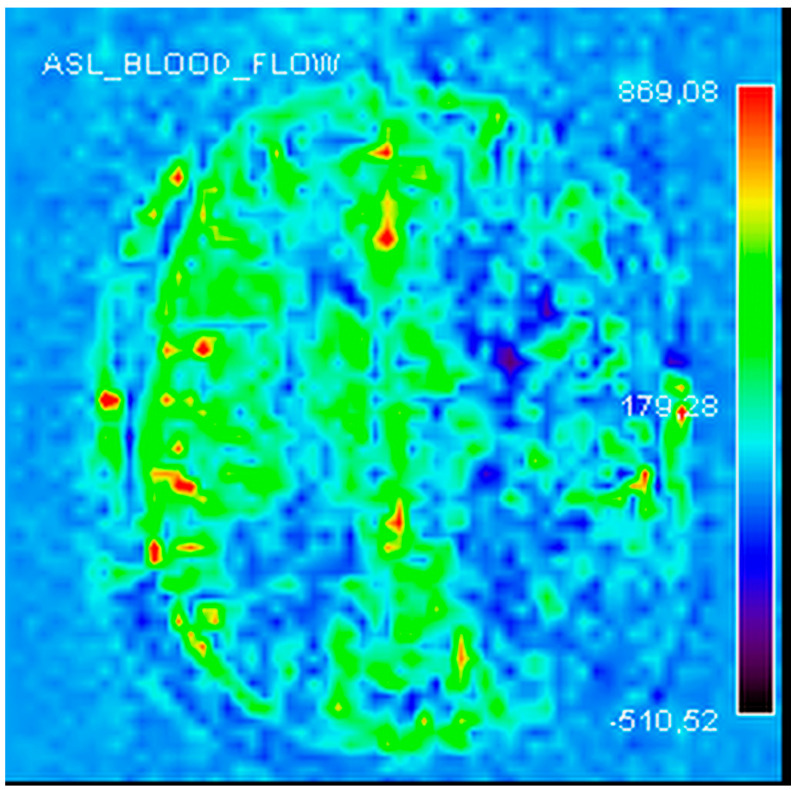
Left cerebral perfusion asymmetry in an MRI perfusion sequence—ASL mapping (arterial spin labelling).

Finally, cerebral perfusion magnetic resonance imaging (MRI) confirmed the presence of a chronic left cerebral perfusion defect (Figure 4).

Taken together, these findings suggest the diagnosis of a transient malperfusion of the aortic arch due to a tear in the residual intimal flap.

**Figure 5:**
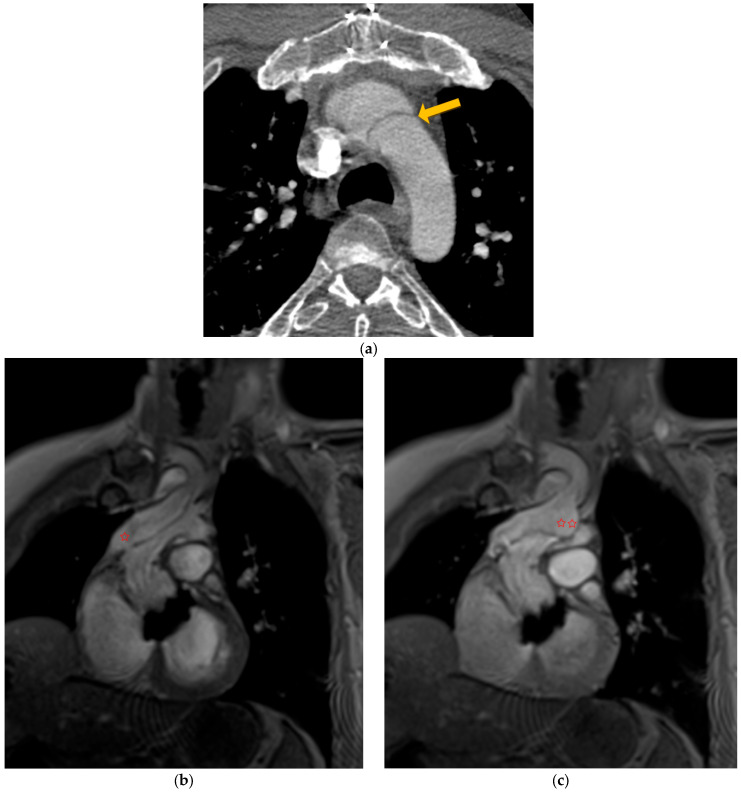
Computed tomography angiography (yellow arrow shows residual intimal flap (**a**) and flow MRI (**b**,**c**) showing a tear in the residual flap (✩) leading to a picture of transient obstruction of the aortic arch; MIR reconstruction (✩✩)—video on annexe.

In this context, dynamic magnetic resonance imaging angiography of the aorta showed complete occlusion of the aortic arch downstream of the innominate artery by the residual intimal flap (Figure 5a, arrow). This confirmed the diagnostic hypothesis of intermittent aortic occlusion (Figure 5b,c and Video S1).

The patient was treated with vascular surgery consisting of prosthetic replacement of the distal part of the aortic arch included reimplantation of the innominate artery. The procedure was successfully performed without complications and resulted in resolution of symptoms.

**Figure 6:**
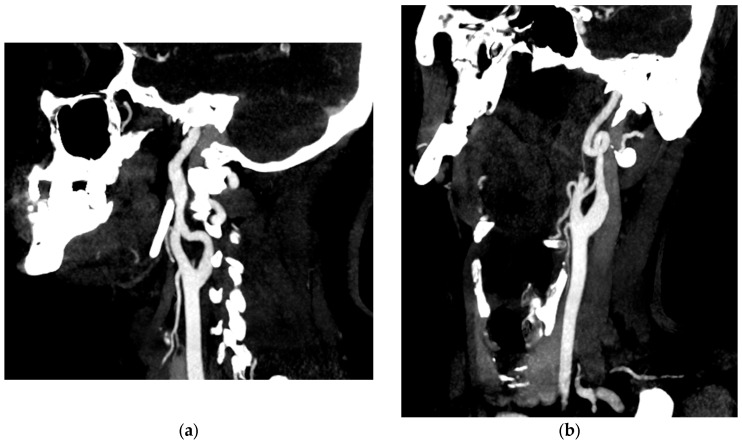
Angioscan of the supra-aortic trunks showing an irregular appearance of the right internal carotid artery (**a**) and a complete loop of the left internal carotid artery (**b**) in its pre-petrous portion.

Etiologically, this patient presented with type A aortic dissection due to a pathological aortic aneurysm before the age of 40 years. There was no associated aortic bicuspidation or significant family history. However, review of the original CTA revealed additional medium calibre arterial anomalies. Ectasia was observed in the right vertebral artery (measured at 7.4 mm) and bilateral common iliac arteries (measured at 16 mm). Arterial tortuosity of the internal carotid artery and plication of the distal left internal carotid artery were also noted (Figure 6). We therefore decided to carry out a gene panel analysis even though there was no phenotypic evidence of Marfan syndrome or vascular Ehlers–Danlos syndrome.

A sequence variation in the exon 01 of the LOX gene has been identified in the heterozygous state. This variation (p.Tyr190*) introduces a premature stop codon. This LOX gene variant detected in our patient has never been described in the literature and is classified as pathogenic (class 5) according to the ACGM classification [5].

The patient is currently being followed in a centre of excellence for rare vascular diseases. His antihypertensive and anticoagulant treatment is continued and he is followed up every six months.

He is in good health and able to return to work. There have been no recurrences since his surgical treatment. A medical genetics consultation has been arranged for first-degree relatives to carry out family screening.

## 3. Discussion

Acquired coarctation is a rare condition. The peculiarity of our presentation was the dynamic and intermittent nature of this intermittent obstruction. To the best of our knowledge, this is the first case described in the literature. The patient presented with symptomatic paroxysmal anisotension which revealed an intermittent dynamic obstruction of the aortic arch. This was a late complication of a type A aortic dissection treated by a Bentall operation some years previously. The rupture of the residual intimal flap downstream of the brachiocephalic trunk and floating in the circulation was responsible for this intermittent coarctation. This was an unusual late complication. The most common late complications of type A aortic dissection are downstream extension of the dissection and aneurysmal progression [3]. The incidence of aortic dissection is estimated to be 5 to 30 cases per million people per year, with men being more commonly affected. Most dissections occur between the ages of 50 and 70, although patients with genetic abnormalities such as Marfan syndrome, Loeys–Dietz syndrome and vascular Ehlers–Danlos syndrome are affected at a younger age [2]. In fact, the International Registry of Acute Aortic Dissections (IRAD) has shown that patients presenting with aortic dissection (AD) before the age of 40 are more likely to have Marfan syndrome or coarctation of the aorta [6]. The natural history and choice of treatment depend on the cause and location of the TAA [2]. Of all TAA, aneurysms of the aortic root, ascending aorta, or both are most common (~60%), rather than those of the descending aorta (~30%) and arch (<10%). Some risk factors are common to both thoracic and abdominal aortic aneurysms, such as hypertension, smoking and hypercholesterolemia. However, hereditary gene variants are more common in patients with TAA disease. Up to 20% of people with TAA or aortic dissection have a family history of thoracic aortic disease (TAD), with at least one affected first-degree relative [2]. Heritable Thoracic Aortic Disease (HTAD) genetic testing panels include more than 1 of 10 genes that have been confirmed to confer a highly penetrant risk of TAD: FBN1, LOX, COL3A1, TGFBR1, TGFBR2, SMAD3, TGFB2, ACTA2, MYH11, MYLK and PRKG1. These panels also include genes that increase the risk of TAD and/or lead to systemic features that overlap with Marfan syndrome, Loeys–Dietz syndrome, or vascular Ehlers–Danlos syndrome. In fact, some mutations in ACTA2, MYH11, MYLK, PRKG1, and LOX have been confirmed to cause HTAD in the absence of significant features of Marfan syndrome or Loeys–Dietz syndrome [7].

A number of variants have already been described [8]. In our patient, this variation in exon 1 of the LOX gene involves the substitution of a thymine (pyrimidine base nucleotide) for an adenine (purine base nucleotide) at position 570 of exon 01 of the LOX gene. This variation, p.Tyr190*, introduces a premature stop codon that is likely to result in: either rapid degradation of the messenger RNA or the production of a COOH-terminally truncated protein. The LOX (lysyl oxidase) gene encodes a protein of the lysyl oxidase family involved in the cross-linking of collagen fibres and elastin. Diseases associated with a defect in the LOX gene include aneurysms and dissections of the ascending aorta [8]. This LOX gene variant detected in our patient has never been described in the literature and is classified as pathogenic (class 5) according to the ACGM classification [5].

Furthermore, even in the absence of a family history or clear phenotypic evidence, genetic counselling may be important and relevant in cases of ascending aortic dissection or aneurysm before the age of 50, especially if there is associated involvement of medium calibre arteries.

Our observation highlights a second important point. In the presence of unexplained symptoms after a first aetiological evaluation, it seems relevant and necessary to perform dynamic imaging at the time of symptomatology to unmask intermittent invalidating diagnoses.

## 4. Conclusions

We describe for the first time a case of symptomatic paroxysmal anisotension with intermittent dynamic occlusion of the aortic arch due to rupture of the residual intimal flap downstream of the brachiocephalic trunk. This was a late complication of a type A aortic dissection treated by a Bentall operation several years previously. An undescribed variation in the LOX gene was observed in our patient.

## Data Availability

No new data were created or analyzed in this study. Data sharing is not applicable to this article.

## Supplementary Materials

The following supporting information can be downloaded at: https://www.mdpi.com/article/10.3390/jcm13040952/s1, Video S1. Visualization of intimal flap with dynamic obstruction of the aortic arch on MRI.
